# Recent Advances in Hydroxyapatite-Based Biocomposites for Bone Tissue Regeneration in Orthopedics

**DOI:** 10.3390/ijms23179721

**Published:** 2022-08-27

**Authors:** Ileana Ielo, Giovanna Calabrese, Giovanna De Luca, Sabrina Conoci

**Affiliations:** 1 Department of Chemical, Biological, Pharmaceutical and Environmental Sciences, University of Messina, Viale F. Stagno d’Alcontres 31, 98166 Messina, Italy; 2 Department of Chemistry “Giacomo Ciamician”, University of Bologna, Via Selmi 2, 40126 Bologna, Italy; 3 Istituto per la Microelettronica e Microsistemi, Consiglio Nazionale delle Ricerche (CNR-IMM), Ottava Strada n.5, 95121 Catania, Italy

**Keywords:** hydroxyapatite, bone tissue regeneration, osteoinduction, osteoregeneration, biocomposites scaffold, bioceramics

## Abstract

Bone tissue is a nanocomposite consisting of an organic and inorganic matrix, in which the collagen component and the mineral phase are organized into complex and porous structures. Hydroxyapatite (HA) is the most used ceramic biomaterial since it mimics the mineral composition of the bone in vertebrates. However, this biomimetic material has poor mechanical properties, such as low tensile and compressive strength, which make it not suitable for bone tissue engineering (BTE). For this reason, HA is often used in combination with different polymers and crosslinkers in the form of composites to improve their mechanical properties and the overall performance of the implantable biomaterials developed for orthopedic applications. This review summarizes recent advances in HA-based biocomposites for bone regeneration, addressing the most widely employed inorganic matrices, the natural and synthetic polymers used as reinforcing components, and the crosslinkers added to improve the mechanical properties of the scaffolds. Besides presenting the main physical and chemical methods in tissue engineering applications, this survey shows that HA biocomposites are generally biocompatible, as per most in vitro and in vivo studies involving animal models and that the results of clinical studies on humans sometimes remain controversial. We believe this review will be helpful as introductory information for scientists studying HA materials in the biomedical field.

## 1. Introduction

Bone tissue is a hierarchical hybrid nanocomposite where the organic extracellular matrix and the inorganic hydroxyapatite phase are organized in a complex and porous structure. Their ability to self-regenerate and withstand large mechanical loads contributes synergistically to the functions of the bone tissue and its self-healing and remodeling properties [1]. Fixation of bone fractures, correction of deformities, and joint replacement are primary unmet medical needs [2]. Currently, clinical approaches to bone repair involve bone autografts and allografts. Therefore, understanding the different biological and chemical factors of bone tissue can facilitate the development of synthetic bone scaffolds designed with specific compositions and structures to form new tissue [3].

Metallic materials, such as stainless steel, cobalt, chromium, and titanium alloys, are commonly used in orthopedic implants for their processability and good mechanical performance. However, metal surfaces are pseudotumor potentials and can cause hypersensitivity [4], and implants must face wear factors, such as friction, lubrication, and wear. As a result, their metal surfaces can cause the formation of nanoparticles that may disperse into adjacent tissues, causing osteolysis.

Ceramic and polymeric materials are also used for regeneration approaches in orthopedics, increasing wear resistance and reducing the risk of developing osteolysis. Therefore, to overcome the drawbacks of metal implants, calcium phosphate ceramics, similar to bone apatite, are among the most used materials as bone substitutes, potentially mimicking the bone-like structure. Nowadays, ceramic materials are useful as osteoinductive and osteoconductive substitutes for bone remodeling or regeneration, or as coatings to metal prostheses to minimize the micro-movements between bones and implants during physiological loading [5,6]. Since these compounds exhibit good surface properties such as roughness, solubility, and porosity, they can influence osteoblasts’ adhesion and proliferation, promoting osteogenesis [7]. However, not all types of calcium phosphates have the same performance. Some degrade slowly in vivo, while others are less stable; some are osteoinductive, while others are not. Three types of calcium phosphates are predominant in the specialized literature: hydroxyapatite (HA, Ca_10_(OH)_2_(PO_4_)_6_), tricalcium phosphates (TCP, Ca_2_(PO_4_)_3_), and biphasic calcium phosphates [6]. These latter are a combination of HA/TCP in different weight ratios, and their use is based on an optimal balance between the more stable HA phase and the more soluble TCP one. As a result, the material gradually dissolves in the body, promoting new bone formation by releasing calcium and phosphate ions into the biological environment [8].

HA has chemical and structural characteristics similar to the inorganic components of bone and teeth [9] and excellent osteo-conductivity/-inductivity, therefore, it is often employed as a bioactive coating for dental and orthopedic implants. In addition, it promotes the adhesion and proliferation of osteoblastic cells on the prosthetic surface and allows biological fixation between bone tissue and implant [10]. However, HA has poor mechanical properties, such as low tensile and compressive strength. For this reason, specific reinforcing materials are typically added, e.g., collagen [11,12], polyacrylamide [13], and graphene oxide [14], to attain HA composites with improved performances, so far resulting often in lower bioactivity compared to pure HA. Much research has been carried out in the scientific landscape that focuses on developing hybrid biomimetic composites by combining biopolymers and HA. Over the past decade, several ceramic/biopolymeric nanocomposites have been developed and tested as implants in the skeletal system to evaluate mechanical properties and their role in osteoinductivity, osteoconductivity, and biodegradability. A number of syntheses of composite biomaterials using different biopolymers such as collagen (Col), gelatin, chitosan (CS), and/or synthetic polymers such as polylactic acid (PLA), polyglycolic acid, and poly (caprolactone), have been described in the literature in association with HA [15].

A bio-inspired approach to the assembly and mineralization process has often been employed to produce biomimetic bone scaffolds through chemical or biological manipulation [16]. For example, the synthesis of bone substitutes via pH-driven mineralization was obtained by introducing the inorganic nanocrystalline mineral constituents into a biopolymer mold to achieve 3D organization through the mineralization of the fibrillar biomaterial [17]. However, biopolymers alone have generally shown poor mechanical strength and a high degradation rate. For this reason, researchers often have employed crosslinkers as a constructive approach to reach both a controlled degradability and the mechanical strengthening of these composites [18,19]. Among the various crosslinking agents, a few have shown promising results, e.g., glutaraldehyde, 1,4-butanediol diglycidyl ether (BDDGE), genipin, and 1-ethyl-3-(3-dimethylamino propyl) carbodiimide hydrochloride (EDC), each with its pros and cons [18].

Due to their relevance in the bone-regeneration research framework involving orthopedic applications, the focus of this review is on bio-inspired systems based on HA ceramics conjugated with biopolymers and strengthened by crosslinking agents. The HA bioconjugate scaffolds described in this review summarize many relevant studies from the last five years showing a significant scientific impact in regenerative medicine. This literature survey on the preparation of hydroxyapatite biocomposites and their properties as a function of biopolymeric matrices, crosslinking methods, and medical applications in bone regeneration can help scientists studying hydroxyapatite materials in the biomedical field.

## 2. Bioceramics: Calcium Phosphates

Calcium phosphate bioceramics, consisting of calcium HA and TCP, exhibit a chemical composition similar to the mineral phase of bone tissues [8]. They are synthetic minerals generally prepared by sintering processes at high temperatures, eliminating water vapor, and subsequently compacted at high pressure [20]. HA and TCP are widely used in the bone regeneration field due to their osteogenic properties and ability to form bonds with host bone [6]. However, the solubility and stoichiometric quantities of Ca^2+^ and PO_4_^3-^ ions of these ceramics can affect their osteo-regenerative properties. HA and TCP have stoichiometric calcium and phosphate ion ratios that differ from each other [21]. HA is the most stable and least soluble at body temperature, with a stoichiometric Ca^2+^/PO_4_^3-^ ratio of 1.67, very similar to that of bone [7,22]. On the other hand, TCP is stable with a stoichiometric ratio of 1.50 [22]. Since the stoichiometric ratio affects the solubility and re-absorbability of the ceramics, it also affects the release of ions from the composites [23,24].

HA is a natural mineral with excellent osteoconductive and osteointegrative properties, comprising about 50% of the bone weight [6] and having mechanical properties similar to the cancellous bone; it is fragile and weak under tension and shear while resistant to compression loads [8,22] (Table 1). The diameter of HA macropores measures over 100 μm, and their interconnection allows the adhesion, proliferation, and differentiation of osteoprogenitor cells, as well as the revascularization and growth of new bone in vivo. The high Ca/P molar ratio and its crystallinity delay the reabsorption rate of HA, resulting in the release of calcium and phosphate ions and a decrease in the volume of the mineral. This can reduce the volume of bone grafts by about 35% in situ after being implanted [25].

Consequently, HA grafts within the host bone can, in the long run, compromise the intrinsic strength of the bone due to worsening mechanical properties [20]. For this reason, HA is most often used as external coating implants or in sites having low mechanical stress [28]. These criticalities are overcome thanks to the development of nanocrystalline HA, which promotes osteoblast adhesion and proliferation, and the deposition of calcium-containing minerals on the scaffold’s surface [29]. As well, the mechanical performance of nano-HA was improved by incorporating nanomaterials of inorganic nature, such as carbon nanotubes (CNT) [30]. On the one hand, the addition of CNT increased the nanocomposite porosity by about 5%; on the other hand, the resistance to fracture reached a value similar to that of human cancellous bone [31].

Tricalcium phosphate (TCP), particularly the rhombohedral form β-TCP, has a Ca/P ratio of 1.5, lower than HA [6], resulting in denser porous structures that allow for a better fibrovascular invasion and bone replacement. At the same time, TCP exhibits worse mechanical properties. Furthermore, at physiological pH, the implanted TCP partially converts to hydroxyapatite and thus inhibits the resorption of the bioceramic matrix. Therefore, TCP is effective in the treatment of bone defects as a filler but is not suitable as a substitute for bone graft due to its unpredictable biodegradation profile.

Biphasic calcium phosphate is a widely used synthetic ceramic obtained by mixing HA and TCP in different percentages to improve the properties of both minerals. In addition, these mixtures’ dissolution rate and mechanical properties can be controlled by modulating the formulation, both in structural applications and in coatings for bone implants [32].

## 3. Polymer-HA Biocomposite Scaffolds

Although hydroxyapatite has osteogenic and osteoconductive properties, it is a very brittle material and is challenging to manufacture in different shapes and sizes [24]. Therefore, HA is often combined with synthetic or natural polymers to mimic the natural environment of the bone, allowing better integration of the prosthesis or enhancing its mechanical properties [33]. As well, combining HA with biodegradable polymers makes it possible to obtain better biocompatibility for the resulting composite, with increased osteoconductivity and osteoinductivity [34]. This section summarizes the biocomposite scaffolds made by combining HA with different natural and synthetic polymers and their effect on bone regeneration, the articles being classified according to the polymeric component present in greater concentration when a mixture of polymers is concerned. Some recent results are also presented in Table 2.

### 3.1. Natural Polymers

**Figure 1:**
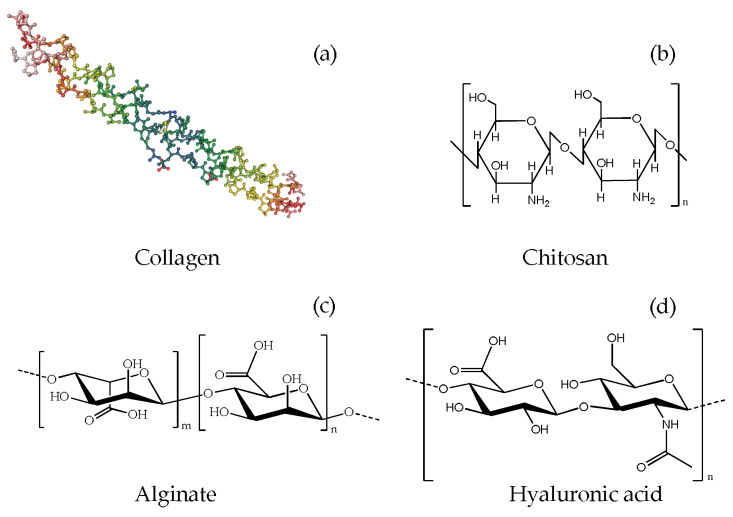
Chemical structure of (**a**) Col [57]; (**b**) CS; (**c**) Alg; (**d**) HyA.

Natural polymers such as Col, CS, Alg, and HyA are used in bio-regeneration studies, including bone regeneration (Figure 1). These biopolymers are suitable for biological applications due to their biocompatibility, high similarity with the extracellular matrix (ECM), and biological tissue structure.

#### 3.1.1. Collagen

Collagen (Figure 1a), extracted from various animal tissues, has been employed in multiple biomedical applications due to its high biocompatibility and good degradability [58]. Its polypeptide chain is rich in glycine and proline aminoacids and is arranged in an α-helical secondary structure. The helices are then arranged in tropocollagen units, a triple right-handed helix stabilized by covalent and non-covalent interactions, which are the structural constituents of collagen self-assembled fibrils.

Bone consists mainly of type I collagen as a biopolymeric component and HA as an inorganic component. When combined, Col and HA can enhance osteoblast differentiation [59,60]. The literature has reported that Col has excellent characteristics in terms of biocompatibility, degradation, and interaction with cells and other biomolecules within our organism [59,61]. The addition of Col to porous HA scaffolds increased their mechanical strength, causing a reduction in porosity [59,62]. The improvement in the mechanical properties has been attributed to the formation of intermolecular H-bonds between Col and HA that increases the breaking energy. Moreover, osteogenic differentiation increases thanks to the intrinsic bioactivity of the HA. As demonstrated by in vitro studies, Col-HA biocomposites show greater cytocompatibility than pure Col scaffolds. In fact, different cell lines such as osteosarcoma cells [39], osteoblast cells [40], and fibroblast cells [43], when exposed to variable amounts of HA in the scaffolds, demonstrate better attachment and proliferation. Greater torsional strength was observed in rabbit models implanted with Col-HA biocomposites in tibial defects compared to β-TCP controls, highlighting the positive effect of the former on bone mechanical properties [63]. Meagher et al. investigated the effect of increasing the HA volume fraction on in vivo angiogenesis and osteogenesis in Col-HA scaffolds after subcutaneous ectopic implantation in mice [64]. Christensen et al. [65] tested the validity of a scaffold consisting of collagen I and MgHA on ten patients with osteochondral lesions during a follow-up period of 1 to 2.5 years. In contrast to previously published results on in vivo experiments carried out on both animal models and clinical cohort studies, the scaffolds showed a poor ability to induce osteochondral regeneration.

Xing et al. investigated the structural features of Col-chitin-HA composites. The results suggested that HA improved their compressive strength and promoted collagen deposition and new bone formation [37]. In another study, Col-nanohydroxyapatite (nHA) and GO composites were prepared, showing good antibacterial properties and biocompatibility [39,66]. However, the clinical results of Col-HA applications seem controversial. Although several variables might influence the results, it has been reported that one of the factors affecting osteochondral regeneration could lie in the area of application of the composite in the body [67].

#### 3.1.2. Chitosan

Chitosan (Figure 1b) is a natural polymer obtained from the partial deacetylation of chitin under alkaline conditions. It is a copolymer consisting of glucosamine and *N*-acetyl glucosamine, forming a linear chain connected by β-1→4 bonds.

Chitin is a nitrogen-rich polysaccharide commonly found in the hard exoskeletons of crustaceans, insects, and other arthropods [68] and, together with its derivatives, is widely used for its biocompatibility, biodegradability, and intrinsic antibacterial activity. Chitin-derived polymers can be easily processed into porous scaffolds or hydrogels, in their pristine form or chelated to different metal ions, thus implementing their antibacterial activity [69]. In a study by Li et al., CS was combined with HA in a scaffold having a hierarchical pore structure. Under the synergistic effect of HA and CS, the scaffolds achieved 277.6% cell viability compared to the pure CS scaffold [44].

CS-HA composites have been often combined with other biopolymeric materials. A study by Shi et al. designed a gradient scaffold obtained using dopamine-modified Alg, HA, and CS [43]. These in vitro studies yielded low cytotoxicity and excellent osteogenic activity that could effectively promote bone regeneration and accelerate bone defect repair in vivo. CS was also integrated into HA scaffolds with Alg in the study of Liu et al., aiming for bone regeneration applications [51]. Türk et al. investigated functionalized CS-Col-MWCNT-HA composite scaffolds obtained by lyophilization, observing low cytotoxicity, high bioactivity, and biocompatibility within in vitro studies [35].

By freeze-drying, Hu et al. developed a biomimetic hybrid scaffold composed of hyaluronic acid, chondroitin sulfate, CS, and nHA. The results showed that these nanohybrids had hierarchical micro/nanostructures and improved osteoblast proliferation and differentiation [56].

#### 3.1.3. Alginate

Alginic acid is a biopolymer extracted from the cell walls of brown algae, consisting of the copolymerization of glucuronic acid and mannuronic acid joined by α-1,4-glycosidic bonds [70]. Alginates (Alg, Figure 1c) are thickeners and stabilizers that form partially water-soluble hydrogels. They are biodegradable and biocompatible materials used for bone regeneration, wound healing, and mechanical properties improvements [46,71].

The Alg-HA scaffold properties vary with the preparation method and the percentages of alginate and HA used. The density of the scaffold increases as the alginate concentration increases, while the porosity decreases because of a parallel increase of viscosity, limiting the diffusion of Alg into the pores [47]. The distribution of Alg inside the porous HA occurs through the interaction between the Ca^2+^ ions of the inorganic matrix and the -COO^-^ groups of the biopolymer, this reticulation leads to an improvement of the mechanical properties of the scaffold [71]. In general, the Alg coating is hydrophilic, resulting in increased swelling and water absorption of a scaffold, whereas crosslinking Alg with Ca^2+^ ions reduce hydrophilicity and, thus, swelling. In a study by Mahmoud et al., Alg-HA scaffolds were shown to induce local bone healing without damaging liver or kidney functions [47].

The degree of Alg gelation and crosslinking are critical factors for controlling the rheological properties, e.g., scaffolds’ printability during the 3D printing process [48]. Ocando et al., prepared alginate and Mg-doped HA scaffolds using “click” chemistry to mimic highly porous structures with the dimensional hierarchy of bone tissue. Uniform dispersion of MgHA on the surface of the pore walls allows for suitable attachment and proliferation of preosteoblast cells [46].

Patil et al. prepared 3D porous scaffolds of HA-coated with Alg-CS by wet chemical precipitation and freeze-drying methods. The pore size of these scaffolds ranged from 30 to 280 μm, and the porosity decreased with increasing HA content, with a parallel increase in their mechanical strength. The scaffolds also showed good swelling behavior and biodegradation. They also supported in vitro attachment and proliferation of MG63 osteosarcoma cells, the HA coating improving the scaffold biocompatibility by modifying its surface roughness and microtopography, which helped increase osteoblast adhesion and migration [50].

Kohli et al. combined Alg with fibrin to produce porous, crosslinked, slowly biodegradable scaffolds with calcium phosphate. MC3T3-E1 cells were tested, which adhered to the scaffolds, proliferated, migrated, and differentiated along the osteogenic pathway during the culture period [49].

In general, Alg-HA scaffolds have shown good physico-chemical and rheological properties, as well as very good biocompatibility, with cell growth and proliferation times adequate for clinical applications. Still, more investigations are needed to optimize their physico-chemical properties further to make them good candidates in tissue engineering applications for bone filling.

#### 3.1.4. Hyaluronic Acid

Hyaluronic acid (HyA, Figure 1d) is an essential component of the extracellular matrix in the human body. In the last decades, it has been widely used in bone regeneration, particularly in the craniofacial and dental fields. Composite scaffolds soaked in HyA have shown excellent potential in improving osteogenesis and mineralization. HyA derivatives were employed as local release vectors rather than scaffold components by loading different osteoinductive or osteogenic factors and getting a controlled release. Such loaded vectors, immobilized on the implant surfaces, are also effective in improving osteointegration: Kaczmarek et al. prepared scaffolds based on HyA, CS, and Col supplemented with nHA by lyophilization, verifying their biocompatibility [72]. Cell culture studies revealed an improved cell attachment and growth on the scaffolds when enriched with nHA, whereas in vivo tests on the tissues surrounding the scaffolds 6 months after implantation indicated a general good wound healing and lack of inflammation caused by the implants. The nHA addition to the HyA/CS/Col scaffolds delayed the implant biodegradation process producing a scaffold with good stability towards contact with surrounding tissues [41].

Yang et al. developed an injectable HyA-Alg hydrogel system embedded in HA and combined this with exosomes, nanovesicles naturally secreted by cells, to repair bone defects in rats in vivo, showing great potential in bone defect regeneration [73]. In the study conducted by Sujana et al. [54], biocompatible nanofibers of HyA, poly(L-lactic acid)-co-poly(ε-caprolactone), fibroin, and HA were fabricated through electrospinning to mimic native ECM. The nanofibrous scaffolds have a higher porosity than those made up of micro-sized fibers and, therefore, an optimal exchange of nutrients and metabolic waste. Osteoblasts grown on these scaffolds showed a 53% higher proliferation level than microfibrous ones and a 63% higher osteogenic differentiation and mineralization thanks to the inclusion of bioactive molecules, demonstrating the good potential as biocomposites for bone tissue engineering [54].

### 3.2. Synthetic Polymers

The use of natural polymers in the production of scaffolds for bone regeneration is limited by several critical issues, such as probable immunological reactions, high costs, and improvable mechanical properties [33]. Therefore, HA is often combined with synthetic polymers to overcome these critical points [24], with the significant advantage of having the possibility to synthesize polymers with characteristics modulable according to the needs. The production of a great variety of bio-inert or bioactive polymeric materials with desired sizes and shapes allows to finely tune the chemical and physical properties, such as acidity, solubility, resorbability, and degradability of the polymer or of its combination with molecules to stimulate bone regeneration. Some examples of synthetic polymers commonly used in tissue regeneration applications are, among others, poly-ε-caprolactone (PCL), polylactic acid (PLA), and polyhydroxybutyrate (PHB) [74]. Regardless of the polymers used to overcome hydroxyapatites’ brittleness and poor moldability, growth factors are generally required to promote osteoinductive properties. Another factor that influences biodegradability and biocompatibility is the polymer’s molecular weight, which must be chosen carefully to match the requirements of bone regeneration processes. Some of the most recent research works are shown in Table 3. Given the vastity of the polymer types that may be found of use in regenerative medicine applications, this review will obviously focus on a limited number of examples amongst the most efficient and used in this field of research, giving only a general idea of the synthetic approaches to scaffolds production [24].

#### 3.2.1. Poly-ε-caprolactone

**Figure 2:**
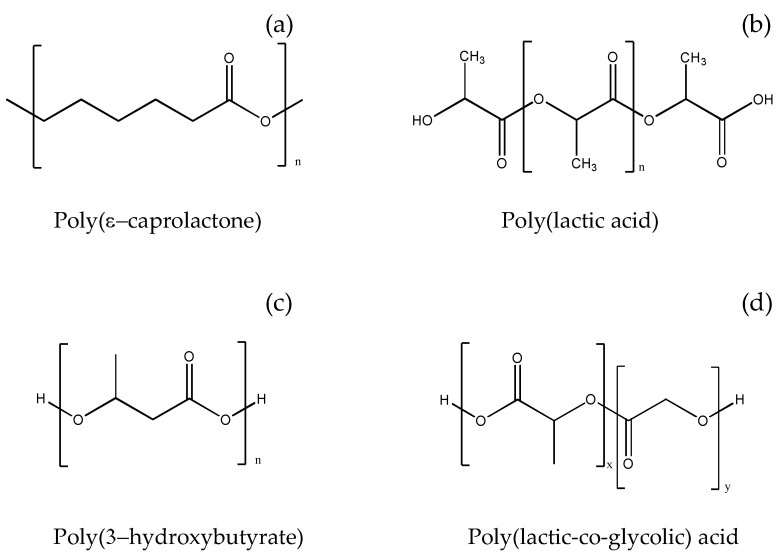
Chemical structure of (**a**) PLC; (**b**) PLA; (**c**) PHB; (**d**) PLGA.

Composites consisting of polymers combined with HA have higher mechanical strength, improved structural integrity, and flexibility than pure polymers. Among the known synthetic polymers used in the preparation of bone grafts, poly-ε-caprolactone (PCL, Figure 2a) represents an attractive alternative as a biomaterial due to its biocompatibility, stability, better performance, shelf life, and cost-effectiveness [76]. PCL is an aliphatic semi-crystalline, bioresorbable polymer, which has been extensively explored as tissue engineering scaffold material because of its slow biodegradation (2–4 years). Due to its low melting temperature (55–60 °C), it can also be easily molded into the desired scaffold design using different fabrication procedures. However, PCL is hydrophobic, providing a lack of wettability and poor cell attachment. Its blending with bioceramic nanofillers substantially overcomes these limits, significantly improving overall biomaterial performance [54].

PCL has been approved by the US Food and Drug Administration for use as an implantable material [100], and its HA composites have been prepared using different methodologies. The mechanical properties of PCL-HA scaffolds depend on the HA volume in the composite, observing an increase in their elastic modulus from 299.3 MPa to 498.3 MPa when the volume of HA increased from 0% to 30% [101]. The addition of HA to PCL can also influence the latter in vitro behavior. Lin et al. developed membranes for bone regeneration based on PCL and cobalt-substituted HA (CoHA). Culturing osteoblast cells on these membranes significantly improved cell proliferation, and the production of calcium deposits also increased by more than 90% compared to PCL alone after seven days of culture [78]. PCL offered limited antimicrobial activity, and the presence of CoHA powder also provided a good antibacterial effect.

In vitro studies on bone regeneration by Shor et al. on a porous 3D PCL-HA scaffold demonstrated improved cell viability and proliferation of primary fetal bovine osteoblasts compared to stock PCL scaffolds [75]. Alkaline phosphatase activity measurement on PCL-HA scaffolds showed a higher cell differentiation than on PCL scaffolds during the differentiation time.

#### 3.2.2. Poly(lactic Acid)

Poly(lactic acid) (PLA, Figure 2b) is obtained from the polyesterification reaction of lactic acid, which, having a chiral center, can polymerize in four different forms: poly(L-lactic acid) (PLLA), poly(D-lactic acid), poly(D, L-lactic acid), and meso-poly(lactic acid) [102]. In general, PLA exhibits high tensile strength and elasticity, however, these properties vary significantly with its different stereoisomers.

Carfì Pavia et al. produced porous PLLA-HA composite scaffolds via thermally-induced phase separation, testing different PLLA-HA weight ratios against cell cultures to evaluate the effect of HA on osteoblastic cell proliferation and differentiation, showing a more significant alkaline phosphatase activity on composite scaffolds than in pure PLLA ones [82]. Additionally, Prakash et al. investigated the mechanical resistance and in vitro bioactivity of porous scaffolds obtained by combining HA and PLA. In vitro analysis showed excellent osteoplastic cells’ growth, proliferation, and differentiation, and mechanical tests demonstrated how these scaffolds are mechanically reliable [86]. Zhang et al., fabricated PLA-HA composite scaffolds by 3D printing, and a rabbit model was established for prefabricating engineered bone with vascularized tissue. After four and eight weeks, neovascularization and bone tissues were analyzed by studying related genes, micro-computed tomography (Micro-CT) images, and histological samples, demonstrating successful scaffold-induced tissue vascularization in vivo [87].

Flores-Sànchez et al. produced electrospun porous matrices with osteoconductive properties by combining the biodegradable PLA, HA, and plasma-polymerized pyrrole, where the constituting nano- and microfibers contained about 35.7% by weight of the inorganic component. The cell viability test demonstrated enhanced cell proliferation due to polypyrrole adhesive properties [84]. Kaito et al. produced a new delivery system for bone morphogenic proteins (BMPs), consisting of an interconnected porous PLA-polyethylene glycol-HA matrix. They obtained the induction of bone regeneration and osteoconduction by releasing BMPs from the biocomposite providing good mechanical support. At eight weeks post-implantation new bone formation and the complete restoration of large bone defects were observed [103]. Additionally, Fernández-Cervantes et al. used 3D printing to produce a microporous composite of polylactic acid, sodium alginate, and HA. This biomaterial showed a density and microporosity similar to that of natural bone and, after treatment with simulated body fluid, exhibited a mechanical resistance to compression greater than that of native bone due to the induced mineralization of HA crystals on its surface [83]. Salehi et al. developed an erythropoietin-releasing PLA-nanoclay-nHA scaffold using the thermally induced phase separation technique to favor bone tissue regeneration. The scaffolds showed good biocompatibility in vitro, while in vivo experiments showed good regenerative capability and new vascularization after eight weeks from implantation [85].

#### 3.2.3. Poly(3-hydroxybutyrate)

Poly(3-hydroxybutyrate) (PHB, Figure 2c) is a crystalline polyester belonging to the family of polyhydroxyalkanoates obtained via enzymatic synthesis by bacteria. PHB degrades in vivo to D-3-hydroxybutyrate, a non-toxic and biocompatible product [94]. Due to its brittleness, PHB is often copolymerized with poly(hydroxyvalerate) to improve processability, whereas reinforcing PHB scaffolds with nHA showed varying effects on the mechanical properties of the matrices. Namely, introducing a 5% by weight of HA nanoparticles into the PHB matrix gave the composite maximum mechanical strength and elastic modulus, whereas the addition of nHA greater than 10% caused a decrease in the mechanical strength of the composite [93].

P3HB-nHA scaffolds showed a better ability to promote cell proliferation and differentiation of osteoblast cells than P3HB scaffolds without nHA. In more detail, cell viability and proliferation increased over time for both matrices, but P3HB-nHA loaded with bone marrow cells exhibited the best results after subcutaneous implantation in a rat model [104]. The implants were covered with a thin layer of connective tissue 45 days after implantation. Internal growth of healthy connective tissue consisting of osteoblasts, macrophages, and mature capillaries was observed in the pores of the scaffolds, indicating active bone formation in the areas adjacent to the implant site and the ability of these implants to support bone regeneration [104]. Although P3HB-nHA scaffolds display these good biological properties, their mechanical strength is poor due to the fragility of both P3HB and HA, so the concerns about their long-term mechanical stability cast doubt on the fact that this composite is a good choice for implantable materials [102].

Chen et al. prepared thin films of P3HB-nHA by electrospinning observing that bone marrow mesenchymal stem cells exhibited better adhesion, proliferation, and osteogenic phenotypes compared to P3HB-only ones. In addition, ex vivo histological analyses revealed both the formation of osteoid tissue and the formation of blood vessels throughout the scaffold after two months from implantation [89]. Degli Esposti et al. prepared bioactive and bioabsorbable porous scaffolds for bone tissue regeneration based on P3HB and HA. HA particles were generated in situ, obtaining composite materials with improved porosity without any degradation of the polymeric matrix. Conversely, samples prepared by the ex-situ method yield suppressed porosity, limiting the amount of HA that could be loaded into P3HB and reducing the resulting bioactivity. These composites were cytocompatible and capable of supporting the adhesion and proliferation of pre-osteoblastic murine cells. In all P3HB-HA scaffolds, cell morphology investigations revealed the presence of differentiated cells with a predominance of osteocyte-like morphology, which was not observed in P3HB-only scaffolds [90]. Cavalcante et al. and Senatov et al. produced P3HB composites with nHA at different concentrations to evaluate microstructural, physical, and biological properties in vitro. Their results indicated that physical properties such as hardness and wettability increased with HA content, and scaffolds containing the nanohybrids exhibited higher cell viability and adhesion [92].

Volkov et al. investigated the osteogenic capability of hybrid composite P3HB-Alg-HA scaffolds on mesenchymal stem cells (MSCs), to regenerate large radial parietal bone defects in rats. Their data demonstrated that this material supported MSCs growth and induced osteogenic differentiation in vitro. CT and histological analyses of P3HB-Alg-HA scaffolds seeded with MSCs after 28 days of implantation in vivo presented a 3.6 times higher regeneration ability compared to MSCs-free scaffolds [91].

#### 3.2.4. Poly(lactic-co-glycolic Acid)

Poly(lactic-co-glycolic acid) (PLGA, Figure 2d) is a copolymer used in many therapeutic devices approved by the FDA, thanks to its biodegradability and biocompatibility. PLGA is prepared by ring-opening co-polymerization of the two cyclic 1,4-dioxane-2,5-dione dimers of glycolic acid and lactic acid [105].

Both poly(lactic acid) and poly(glycolic acid) are characterized by low mechanical strength and, in their polyanionic form, they exhibit a lower local pH than the bulk solution. For these reasons, the researchers copolymerized the two acids, modulating their ratios and overcoming the above-mentioned critical issues [106]. After degradation, PLGA breaks down into lactic acid and glycolic acid, which are byproducts of human metabolism and can be excreted from urine. PLGA systems are primarily employed in drug delivery, but they are also used in the regeneration of bone, skin, cartilage, and nerves [105,107]. Dos Santos et al. developed bilayer membranes with a dense layer of PLGA-HA and an electrophilic layer of PLGA and HA/β-TCP. The membranes showed a degree of porosity of 38.2%, preventing fibroblast infiltration but allowing the migration of osteoblasts and the permeation of nutrients. The mass loss due to in vitro degradation was only 10% after 60 days, a profile suitable for the application requirements [95]. Ceccarelli et al. analyzed the osteoconductivity ability of two different PLGA-based scaffolds alone or combined with HA, versus stem cells derived from the periosteum, both in vitro and in vivo. Their results demonstrated that PLGA/HA scaffolds were osteoconductive in vitro and able to promote bone healing in vivo [99]. Fu et al. fabricated a two-layer membrane based on PLGA and HA by phase inversion for the dense layer and electrospinning for the porous layer. The results showed that a dense layer of PLGA with 5% HA could meet the mechanical strength requirements and have excellent barrier function even in post-degradation conditions. Additionally, a porous layer consisting of PLGA and nHA (in a 7:3 ratio) could achieve good physical/chemical properties and improve mineralization in vitro, providing superior cell adhesion, proliferation, and differentiation capabilities [98]. Jin et al. fabricated by electrospinning a nano-fibrous membrane of fish Col and nHA enhanced with PLGA for guided bone regeneration. These membranes showed favorable cytocompatibility with bone mesenchymal stem cells (BMSCs) and human gingival fibroblast cells [38]. Yan et al. prepared electroactive microspheres by immobilizing an aniline tetramer on PLGA-HA microspheres. These electroactive, biodegradable, and injectable microspheres were successfully fabricated using a high-voltage electrostatic technique and the oxidative polymerization of DOPA [98]. The microspheres could act as an injectable biomaterial scaffold to support cellular adherence and proliferation. In vitro studies demonstrated that the electroactive microspheres facilitated cell proliferation, osteogenic differentiation, and the expression of osteogenic markers through enhancing cellular signaling. Besides, the combination with DOPA could promote osteogenic differentiation, and in vivo results demonstrated that the microspheres effectively repaired rat calvarial defects. The successful regeneration of bony tissues was confirmed by mineralized collagen depositions and enhancement of bone content in the defect area, further indicating the significant potential for bone repair and regeneration applications at a clinical level [96]. Lu et al. prepared an HA-PLGA composite scaffold loaded with doxorubicin (DOX) and coated with polydopamine (PDA) to achieve the dual functions of bone tumor inhibition and bone repair. It was found that the PDA coating improved hydrophilicity and mechanical properties and led to a more sustained drug release. The DOX @ PLGA-PDA-HA scaffold significantly inhibited tumor cell growth and enhanced osteoblast adhesion and proliferation. Furthermore, the PDA coating improved the bioactivity of the scaffold, as suggested by biomineralization in vitro [97].

## 4. Crosslinking Methods and Agents in HA-Based Composite Scaffolds

The different polymers employed in preparing HA-based scaffolds for tissue engineering applications often have the disadvantage of conferring low mechanical strength and high solubility in aqueous solutions, rapidly deteriorating in the cell culture environment [64]. Since the beginning of biomaterials fabrication, various crosslinking methodologies have been established, compatibly to the chemical nature of the organic and inorganic matrices, to limit these drawbacks and keep the mechanical properties of the composite constant [108,109]. Crosslinked polymers have a higher modulus of elasticity (Young’s modulus) and a lower swelling degree than the non-crosslinked ones. Thus, the crosslinking of the material is essential to regulate the composites’ mechanical properties and degradation time. However, from a practical point of view, a composite should be manufactured using minimal solvents or chemicals and still have finely adjustable properties to meet different requirements in terms of a size range, mechanical strength, porosity, and degradation time [110].

The following section describes some of the crosslinking agents most recently used in preparing HA-based scaffolds for bone regeneration, classifying the methods into four categories: physical, chemical, enzymatic, and non-enzymatic [111].

### 4.1. Physical Biopolymers Crosslinking

Physical crosslinking processes rely on external high-energy (thermal or radiation) sources to create an excited species that can decompose and create organic free radicals [112]. These methods include dehydrothermal treatment (DHT) and irradiation with ultraviolet (UV) or gamma radiation. In general, physical crosslinking is considered potentially non-toxic, but the often-resulting dehydration of the scaffold surface makes the produced materials less favorable for cell culture applications.

#### 4.1.1. Dehydrothermal Treatment

**Figure 3:**
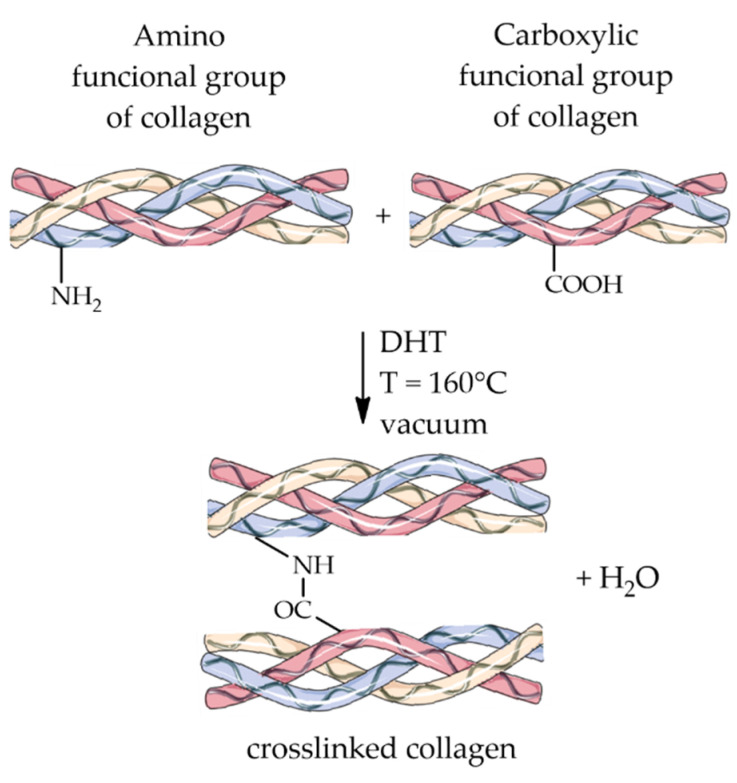
Schematic representation of the crosslinking reaction through dehydrothermal treatment.

DHT is a physical approach in which the biopolymer is subjected to a temperature greater than 160 °C under a vacuum [113] and where crosslinking occurs thanks to the removal of water molecules following heat treatment and the consequent formation of intermolecular bonds. Often, this crosslinking process results from bonding amino and carboxyl groups of a protein when they are spatially close, as illustrated for collagen in Figure 3.

A positive side effect of DHT is that exposure to high temperatures sterilizes the material, reducing the immunogenic response and increasing cellular activity [114]. Therefore, the DHT method is widely used in tissue engineering applications primarily for its non-toxic effects [115]. Kozlowska et al. reported the positive effect of combining DHT with the addition of a mixture of 1-ethyl-3(3-dimethylaminopropyl) carbodiimide (EDC) and N-hydroxysuccinimide (NHS) on the physicochemical properties of Col-HA materials [116]. In addition, some recent studies used DHT to prepare HA scaffolds, where Col-glycosaminoglycan materials were used on soft tissues [113], and biocompatible Col-CS-HA porous composites were fabricated to restore defective maxillofacial mandibular bone [117].

#### 4.1.2. Radiation

The crosslinking induced by ultraviolet (UV) or gamma radiation is a simple and non-toxic method whereby bonds form between aromatic amino acids (such as tyrosine and phenylalanine) of the polypeptide chains inside the protein, leaving the acid and basic side chains free for cell recognition. As for the previous method, this approach also offers the advantage provided by UVC radiation, namely, that emitted by Hg lamps (λ = 254 nm), causing the material sterilization. However, some critical issues are associated with UV treatment, such as the limited penetration of the radiation, which is only effective at depths of microns. Therefore, UV crosslinking is more suitable for thin films or UV-transparent scaffolds and for the photochemical synthesis of biomaterials. Campiglio et al. fabricated sub-micrometric, UV-crosslinked fiber scaffolds for regenerative medicine applications to mimic the extracellular matrix’s morphology and chemistry [118]. Kim et al. prepared a hydrogel of silk fibroin and HA nanoparticles through gamma irradiation treatment. The results revealed that composite hydrogels improved osteogenic differentiation compared to pure silk fibroin ones and demonstrated their great potential in the production of scaffolds for bone tissue engineering where osteogenesis is required [119]. Ghobashy et al. described the preparation of a hydrogel of carbonated HA and poly(sodium hyaluronate-acrylamide) induced by gamma radiation. The hydrogel acted as a HA template and carbon precursor. In vitro studies demonstrated that using this hydrogel as a biocompatible nanomaterial improved osteogenic ability compared with HA [120]. Davidenko et al. prepared collagen- and gelatin-based scaffolds crosslinked through UV irradiation (λ = 254 nm) to modulate the materials’ properties while keeping their biological functionality [121].

### 4.2. Chemical Biopolymers Crosslinking

Chemical crosslinking refers to the intermolecular or intramolecular formation of covalent bonds. It is widely used in regenerative medicine due to its speed, versatility, and great crosslinking performances. The crosslinking reagents, or “crosslinkers,” are bifunctional organic molecules classified into different types, each with its specific function and application, based on factors such as reactivity and spacer length [122]. The development of novel bone-like scaffolds by bio-inspired, pH-driven mineralization with HA can be largely improved by the action of the various crosslinkers that will be discussed in the next section, such as glutaraldehyde (GTA), 1-ethyl-3-[3-dimethylaminopropyl] carbodiimide hydrochloride (EDC) and N-hydroxysuccinimide (NHS), 1,4-butanediol diglycidyl ether (BDDGE), and genipin. These additives significantly promote beneficial enzymatic resistance and swelling ability, modifying the mechanical behavior and cell interactions as a function of the crosslinker. Therefore, by activating specific crosslinking mechanisms, hybrid composites have been designed and tailored to develop tissue-specific biomimetic materials for hard tissue engineering.

#### 4.2.1. Glutaraldehyde

**Figure 4:**
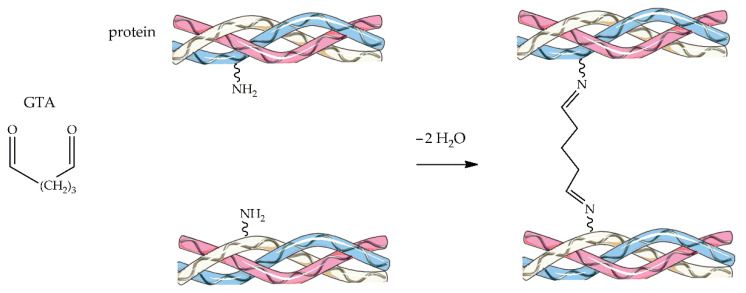
Schematic representation of the GTA-crosslinking reaction.

Glutaraldehyde (GTA) is a bifunctional crosslinking agent that can be efficiently employed with fibrous proteins such as collagen, causing the formation of imino bonds with the amino groups of lysine or hydroxylysine residues to increase protein stabilization (Figure 4). GTA is one of the first crosslinkers reported for biomedical applications, thanks to its low-cost, extensive availability, high reactivity, and strong stabilization capabilities [123]. Nevertheless, the local cytotoxicity of GTA can cause an unwanted response in the host’s immune system, which limits its use in the biomedical field. However, many studies illustrated the cytotoxicity of GTA is concentration-dependent, and using up to 8% GTA is considered safe and non-toxic [124]. GTA toxicity can also be reduced by washing the scaffolds with a glycine solution to remove the unbound aldehyde groups, the actual potentially toxic stimulants [125]. In some examples of bone tissue engineering applications, Salifu et al. prepared gelatin-HA electrospun fiber scaffolds crosslinked with GTA [126], while Iglesias-Mejuto and García-González fabricated bioactive 3D hydrogel scaffolds based on reinforced Alg-HA crosslinked with CaCl_2_ and GTA [127]. In many cases, the scaffold fabrication method involved the freeze-drying technique, with fewer papers focusing on electrospinning procedures, and GTA toxicity was often evaluated by studying cell-material interactions.

#### 4.2.2. 1-Ethyl-3-(3-dimethylamino Propyl) Carbodiimide (EDC) and N-hydroxysuccinimide (NHS)

**Figure 5:**
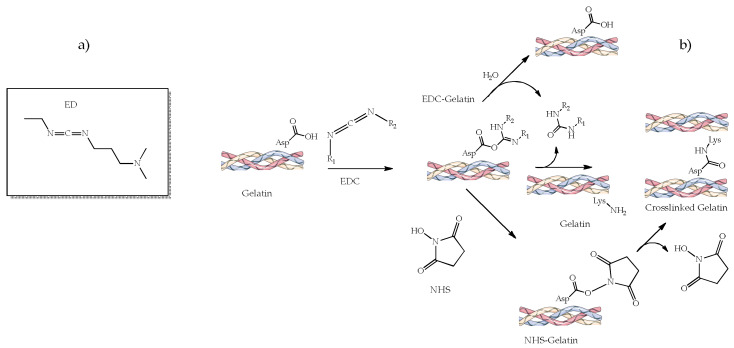
EDC chemical structure (**a**) and schematic representation of the EDC-crosslinking reaction (**b**) with or without NHS.

1-ethyl-3-(3-dimethylamino propyl) carbodiimide (EDC, Figure 5a), often added as hydrochloride salt, is a crosslinking agent commonly employed to conjugate carboxyl or phosphate groups to primary amines. It reacts with any biopolymer to form an active O-acylisourea intermediate that binds the amino groups forming an amide and releasing urea (Figure 5b) [116], which is soluble in water and can be easily eliminated from the body. In fact, unlike GTA or genipin (vide infra), EDC is nontoxic because it is not part of the final product [111].

EDC crosslinking reactivity is pH-dependent, being higher and more efficient under acidic buffer conditions [128]. The reaction can still be compatible with physiological conditions, but the efficiency is relatively low. Another essential aspect of EDC crosslinking is the coupling with N-hydroxysuccinimide (NHS) to improve the stability and efficiency of the crosslinking reaction (Figure 5b).

A common drawback encountered in crosslinking with EDC is that it involves the primary amino groups (on lysine residues) and carboxylate anions (on glutamate or aspartate residues), thus minimizing the availability of essential cell-binding motifs on protein-like biomaterials. Indeed, there is a significant need to reduce the EDC concentration to obtain scaffolds with unhindered cell reactivity without compromising their surface chemistry and biomechanics. Recent studies have documented that a substantial reduction in EDC concentration has significantly improved the scaffolds’ biological performance together with their mechanical properties and chemical stability. Salehi et al. proposed a study on peripheral nerve regeneration using a Col hydrogel containing nHA crosslinked with EDC [128]. They had a high porosity, high swelling properties, and higher values of the compressive modulus compared with non-crosslinked samples. Kozlowska et al. combined dehydrothermal treatment and a mixture of EDC/NHS crosslinking on Col-HA materials improving the physicochemical properties and the stability of the resulting scaffolds [116]. Castilla Bolaños et al. developed porous small intestine submucosa-HA sponges for bone tissue engineering and regeneration [129], whereas Kaczmarek et al. prepared a CS-Coll-HyA-HA sponge for in vitro study on human osteosarcoma SaOS-2 cells [72], both crosslinked by EDC/NHS. In most of the analyzed papers, EDC/NHS has been commonly used as a crosslinker for developing bone-specific scaffolds with gelatin or collagen. Akin to other crosslinkers, the method to fabricate 3D scaffolds mainly used freeze-drying, and all reported in vitro studies were performed using bone-specific cell cultures.

#### 4.2.3. 1,4-Butanediol Diglycidyl Ether

**Figure 6:**
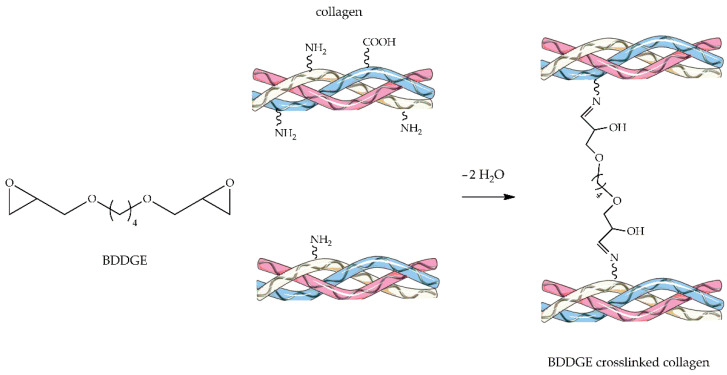
Schematic representation of BDDGE-crosslinking reaction.

1,4-Butanediol diglycidyl ether (BDDGE, Figure 6) is a bifunctional crosslinker often used to stabilize dermal collagen filler. Its crosslinking mechanism with any biopolymer exploits the reactivity of the epoxide groups present at both ends of the molecule, reactivity which heavily depends on pH and temperature conditions. BDDGE can form covalent bridges with macromolecular substrates through nucleophilic substitution reaction [111,130]. The crosslinking mechanism proceeds, for example, through imide formation via epoxide ring-opening by amine groups under physiological pH and subsequent dehydration, as illustrated in Figure 6.

Calabrese et al. reported a synthesis of HA-Col porous scaffolds [60,131,132] reinforced with silver and gold nanoparticles [131,133], satisfying both antimicrobial and osteo-regenerative properties [134]. The scaffolds were prepared by incorporating Col with bioactive magnesium-doped nHA and stabilizing the structure with the highly reactive BDDGE. Sartori et al. developed a new bi-layered scaffold stabilized with BDDGE for osteochondral tissue regeneration [135]. The results showed that chondral and bone scaffold layers represented biocompatible matrices able to sustain hMSCs attachment and proliferation. Furthermore, the ectopic implantation of the engineered osteochondral scaffolds indicated that hMSCs could be colonized in-depth. Sprio et al. developed a multifunctional superparamagnetic hybrid scaffold recapitulating the different features of alveolar bone, periodontal ligament, and cementum by integrating the biomineralization process, tape casting, and electrospinning techniques. The scaffolds could promote osteogenesis and be activated by remote magnetic signals [136].

#### 4.2.4. Genipin

**Figure 7:**
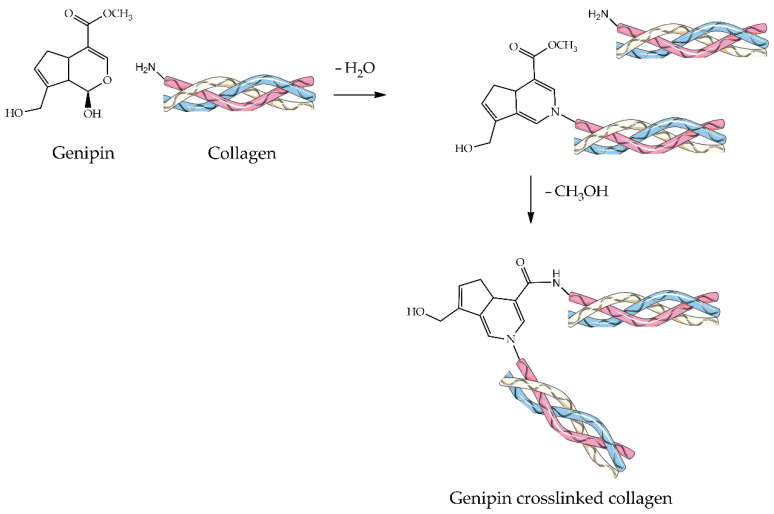
Schematic representation of the Genipin crosslinking reaction.

Genipin (Figure 7) is a natural crosslinker derived from the gardenia fruits that can form permanent intra- and inter-molecular bonds between two protein macromolecules [18]. On the one hand, the crosslinking pathway (Figure 7) starts with a nucleophilic attack of the collagen’s primary amine of lysine, hydroxylysine, or arginine residues on the genipin C3 carbon to form an N-heterocycle. On the other hand, another nitrogen atom of collagen is involved in a nucleophilic acyl substitution of the ester methoxy group forming an amide [137,138]. The intra- and intermolecular crosslinking of collagen with genipin molecules probably induce the disruption of non-native collagen structure [139].

In the last decade, genipin has been widely used as a suitable crosslinker for human tissue engineering applications owing to its natural origin and low immunogenicity, as well as its non-toxic behavior and potential safety [140]. Still, like GTA, its cytotoxicity is largely debated in the literature. Nonetheless, numerous studies have documented that the toxicity of genipin is dose-dependent and acute but not time-dependent. Aiming at bone tissue engineering, Lu et al. developed genipin-crosslinked hydroxypropyl CS-nHA composite implants [141], whereas Zafeiris et al. synthesized 3D hybrid HA hydrogel scaffolds. Additionally in this latter case, chemical crosslinking was performed using genipin to improve the scaffolds’ mechanical properties, while their rheology was modified by employing an acetic acid/gelatin solution [142]. In a study conducted by Scialla et al., genipin-crosslinked Col-HA scaffolds inducing chondrogenesis were evaluated, revealing an isotropic and highly homogeneous pore distribution. In particular, the presence of genipin in “bulk” led to a more uniform and homogenous chondral-like matrix deposition by hMSC [143]. Collectively taken, genipin as a crosslinker is still considered safe and effective if restricted to a minimal dosage. However, there is a need for concentration optimization to eliminate any toxic and undesired responses.

## 5. Conclusions

In bone tissue engineering, calcium phosphate ceramics are favorable materials due to their chemical and morphological similarity to bone structure. The reviewed literature has made it evident that the design of suitable bone substitutes targeting orthopedic applications depends on factors such as the intrinsic properties of the materials and the method of preparation, which significantly affect the applications. Since the inorganic part of the bone is mainly apatite, (nano)hydroxyapatite is the most suitable material due to its similarity to natural bone. Pure HA is, however, poorly osteoconductive and highly fragile, and its osteoconductive capabilities have been often improved by changing the crystallinity, porosity, size, and surface characteristics. Osteoinductivity can be provided by combining HA with ions such as Sr, Mg, and Zn. In addition, HA can be combined with natural or synthetic polymers and crosslinkers to overcome these problems.

Increased micro and macroporosity are able to improve cell-to-cell connection, migration, signaling, differentiation, proliferation, and protein adhesion of the scaffolds. However, it also brings about a reduced mechanical strength of the material. nHA can be used to overcome the weakening of the composite with increasing porosity due to its nanometer size and larger surface area to allow cells to adhere and proliferate. In addition, the use of nHA also improves the bone-forming capacity and the mechanical strength of the newly formed bone.

By evaluating the effects exerted by different polymers and crosslinkers in modifying the performance of hybrid HA composites, this review demonstrated the possibility of fine-tuning the physico-chemical and mechanical characteristics of HA-based biocomposites without compromising their biocompatibility. Many recent studies combining HA with different polymers and the subsequent functionalization of the composites with suitable crosslinkers yield reinforced 3D hybrid scaffolds. The final choice of polymers and crosslinkers to be employed largely depends on the material’s applicability to adapt the properties to tissue-specific applications.

The surveyed literature allows us to conclude that, in general, all the considered crosslinking mechanisms are able to significantly improve the physico-chemical and mechanical properties of the composites compared to the non-cross-linked materials, also favoring the biological performance by improved cell-material interactions. The composites reviewed here have displayed greater enzymatic resistivity, swelling capacity, good mechanical resistance, adequate structural and dimensional properties, as well as satisfactory biological performances. Further investigations on HA-based biocomposites might fruitfully explore in vitro and in vivo tests with longer incubation times to obtain more information on the effects of the different crosslinkers on bone matrix production and the expression of selective markers of the osteogenic phenotype. These studies may offer further insight into the ability to choose and customize HA-based composites for hard tissue engineering applications with bioactive properties.

## Figures and Tables

**Table 1:** Comparison between synthetic CaP and bone mechanical properties.

Material	Compressive Strength (MPa)	Tensile Strength (MPa)	References
Cancellous bone	41.4	3.5	[26]
Porous HA $\frac{\left[Ca^{2+}\right]}{\left[PO_{4}^{3-}\right]}=1.67$	6.9–68.9	2.48	[21]
Porous TCP $\frac{\left[Ca^{2+}\right]}{\left[PO_{4}^{3-}\right]}=1.50$	2.9	N/A	[27]

Abbreviations: HA, hydroxyapatite; TCP, tricalcium phosphates.

**Table 2:** Details of different natural polymers recently used in the preparation of hydroxyapatite biocomposite scaffolds.

Polymer and Additives	Crosslinker	Fabrication Method	In Vitro Study	In Vivo Study	Refs
Col, CS, Multiwalled Carbon nanotubes (MWCNT)	Dehydrothermally (DHT) crosslinked under vacuum for 48 h at 120 °C.	Lyophilization	-	-	[35]
Col	BDDGE 2.5 mM at 4 °C for 19 h.	Lyophilization	hMSCs Human Mesenchymal Stem Cells	Rabbit (lumbar spine)	[36]
Col, chitin	Epichlorohydrin/chitin (10:1 molar ratio) at 60 °C for 6 h.	Lyophilization	MC 3T3 osteoblast precursor cell line	Male SD Rats (tibial defect)	[37]
Fish Col, Poly(lactide-co-glycolide) (PLGA)	N-hydroxysuccinimide (NHS) 10 mM, EDC 10 mM at 4 °C for 24 h.	Electrospinning	BMSCs, HGF	-	[38]
Col, Graphene oxide (GO)	Ribose 0.2 M, acetone 10 wt.%, and ammonia 2 wt.% at rt for 24 h.	Biomimetic mineralization Lyophilization	Osteoblasts	-	[39]
Col, Zinc silicate	Genipin 1 wt.%	3D-printing	BMSC	Rat (critical size calvarial defect)	[40]
Col, CS, Hyaluronic acid (HyA)	-	Lyophilization	-	-	[41]
CS, Polyvinyl alcohol (PVA), 3-aminopropyltriethoxysilane	Citric acid 1.5 wt./v.% at rt for 2 h.	Electrospinning	Fibroblast cells derived from human lung tissue	-	[42]
CS, Alg, Dopamine	CaCl_2_ solution (5 wt.%) for 5 h at rt.	Lyophilization	L929 cells Subclone of parental strain L	Rabbits (femur)	[43]
CS, PVA, PLA	-	Lyophilization	MC3T3-E1 subclone mouse pre-osteoblasts	-	[44]
CS, Sr^2+^, Mg^2+^, Zn^2+^	Genipin 1 wt.% at 37 °C for 12 h.	In situ precipitation	MC 3T3-E1	-	[45]
Furan-modified Alg, Mg^2+^, Poly(propylene oxide)-b-poly(ethylene oxide)-b-poly(propylene oxide) bifunctional maleimide	EDC 8 mM at rt for 1 h.	Lyophilization	MC 3T3-E1	-	[46]
Alg	CaCl_2_ 0.1 M solution at 40 °C overnight.	Lyophilization	-	Rats (cortical bone)	[47]
Alg, PVA	CaCl_2_ 100 mM solution at rt for 1 h.	3D-printing	MC 3T3	-	[48]
Fibrin, Alg	0.2% *v*/*v* glutaraldehyde in ethanol, 2-(*N*-morpholino ethanesulfonic acid solution at rt for 4 h.	Lyophilization	MC 3T3	-	[49]
Alg, CS	CaCl_2_ 1 wt.% solution at rt for 15 min.	Lyophilization	MG63 human osteosarcoma cell line	-	[50]
Alg, CS	CaCl_2_ 15 wt.% solution at rt for 30 min.	Lyophilization	BMSCs	-	[51]
Alg,	D-Gluconic acid δ-lactone, CaCl_2_ 10 mM solution at rt for 1 h.	Lyophilization	BMSCs	-	[52]
Col, CS, HyA	EDC 50 mM, NHS 25 mM in ethanol 98 % at rt for 4 h.	Lyophilization	SaOS-2	-	[53]
Poly(L-lactic acid)-co-poly(ε-caprolactone), silk fibroin, HyA	-	Electrospinning	hFOBs	-	[54]
HyA	-	Lyophilization	hUCMSCs	-	[55]
HyA, CS, Chondroitin sulfate	EDC, NHS (2:1 molar ratio) at rt for 5 h.	Lyophilization	Osteoblasts	-	[56]

Abbreviations: Col, collagen; CS, chitosan; MWCNT, multiwalled carbon nanotubes; DHT, dehydrothermally; BDDGE, 1,4-butanediol diglycidyl ether; hMSCs, human mesenchymal stem cells; SD, sprague dawley; PLGA, Poly(lactide-co-glycolide); NHS, N-hydroxysuccinimide; EDC, 1-ethyl-3-(3-dimethylamino propyl) carbodiimide hydrochloride; BMSCs, Bone mesenchymal stem cells; HGF, human gingiva fibroblasts cells; GO, graphene oxide; BMSC, bone marrow stromal cells; HyA, Hyaluronic acid; PVA, polyvinyl alcohol; Alg, alginate; PLA, poly lactic acid); SaOS-2, human osteosarcoma cell line; hFOBs, human fetal osteoblasts; hUCMSCs, human umbilical cord mesenchymal stromal cells.

**Table 3:** Details of different synthetic polymers recently used in the preparation of hydroxyapatite biocomposite scaffolds.

Polymer and Additives	Fabrication Method	In Vitro Study	In Vivo Study	Refs
PCL	Precision extrusion deposition	Osteoblasts	-	[75]
PCL, ZnO nanoparticles	Electrospinning	Bone-derived MG-63 (human osteosarcoma) cells	-	[76]
PCL, Alg	Electrospinning	hDPSCs Human dental pulp stem cells	-	[77]
PCL, Co^2+^	Electrochemical deposition	MG-63 cells	-	[78]
PCL, poly(glycerol sebacate), Simvastatin	Electrospinning	MC 3T3-E1 cells	-	[79]
PCL	3D-printing	Osteoblast cells	Rats (calvarial defect)	[80]
PCL, MgO	3D-printing	MC 3T3-E1 cells	-	[81]
PLA,	Drying under vacuum	MC 3T3-E1 cells	-	[82]
PLA, Alg	3D-printing		-	[83]
PLA, polypyrrole	Electrospinning	Fibroblast-like cells	-	[84]
PLA, nanoclay	Lyophilization	MG-63 cells	Albino male rats (critical size calvarial defect)	[85]
PLA	3D-printing	BMSCs	-	[86]
PLA	3D-printing	BMSCs	White rabbits (tibial periosteum defect)	[87]
PLA, Silk	3D-printing	-	-	[88]
Poly-hydroxybutyrate (PHB)	Electrospinning	BMMSCs	-	[89]
PHB	Thermally-induced phase separation	MC 3T3-E1 cells	-	[90]
PHB, Alg, mesenchymal stem cells	Hydrogel synthesis	MSCs	Rats (critical size calvarial defect)	[91]
PHB	Solution casting	L929 fibroblasts cells	-	[92]
PHB	Electrospinning	Osteoblasts	-	[93]
PHB	Compression molding	MMSCs	Mice (tibial bone defect)	[94]
PLGA	Electrospinning	MC 3T3-E1 cells	-	[95]
PLGA, 3,4-hydroxyphenalyalanine	High-voltage electrostatic technique	MC 3T3-E1 cells	Rat (calvarial defects)	[96]
PLGA, Polydopamine, Doxorubicin	Electrospinning	MG-63 cells	Mouse (skull defects)	[97]
PLGA	Electrospinning	L929 fibroblasts cells	-	[98]
PLGA	Electrospinning	hPCs Haemopoietic Progenitor Cells	patients (>18 years) requiring monolateral or bilateral maxillary sinus floor augmentation without comorbid disease	[99]

Abbreviations: PCL, poly-ε-caprolactone; Alg, alginate; hDPSCs, human dental pulp stem cells; PLA, poly lactic acid; BMSCs, bone mesenchymal stem cells; PHB, poly-hydroxybutyrate; BMMSCs, bone marrow mesenchymal stem cells; MSCs, mesenchymal stem cells; MMSCs, multipotent mesenchymal stromal cells; PLGA, Poly(lactide-co-glycolide); hPCs, haemopoietic progenitor cells.

## Data Availability

Not applicable.

## Abbreviations

Alg	Alginate
BDDGE	1,4-butanediol diglycidyl ether
BMP	Bone morphogenic proteins
BMSC	Bone mesenchymal stromal cells
BMSCs	Bone mesenchymal stem cells
CNT	Carbon nanotube
Col	Collagen
CS	Chitosan
CT	Computed tomography
DHT	Dehydrothermal
DOX	Doxorubicin
ECM	Extracellular matrix
EDC	1-ethyl-3- (3-dimethylamino propyl) carbodiimide hydrochloride
GO	Graphene oxide
GTA	Glutaraldehyde
HA	Hydroxyapatite
hMSC	Human mesenchymal stem cells
HyA	Hyaluronic acid
MgHA	Magnesium-doped hydroxyapatite
MSCs	Mesenchymal stem cells
MWCNT	Multiwalled carbon nanotubes
nHA	Nanohydroxyapatite
NHS	N-hydroxysuccinimide
PCL	Poly-ε-caprolactone
PDA	Polydopamine
PHB	Polyhydroxybutyrate
PLA	Polylactic acid
PLGA	Poly(lactide-co-glycolide)
PLLA	poly (L)-lactic acid
PVA	Polyvinyl alcohol
SD	Sprague Dawley
TCP	Tricalcium phosphates
UV	Ultraviolet
